# Current and Emerging Strategies for Tubo-Ovarian Cancer Diagnostics

**DOI:** 10.3390/diagnostics13213331

**Published:** 2023-10-28

**Authors:** Mark R. Brincat, Ana Rita Mira, Alexandra Lawrence

**Affiliations:** 1 Department of Gynaecological Oncology, Royal London Hospital, Barts Health NHS Trust, London E1 1FR, UK; 2 Hospital Garcia de Orta, 2805-267 Almada, Portugal

**Keywords:** ovarian cancer, screening, diagnosis, biomarkers, imaging

## Abstract

Tubo-ovarian cancer is the most lethal gynaecological cancer. More than 75% of patients are diagnosed at an advanced stage, which is associated with poorer overall survival. Symptoms at presentation are vague and non-specific, contributing to late diagnosis. Multimodal risk models have improved the diagnostic accuracy of adnexal mass assessment based on patient risk factors, coupled with findings on imaging and serum-based biomarker tests. Newly developed ultrasonographic assessment algorithms have standardised documentation and enable stratification of care between local hospitals and cancer centres. So far, no screening test has proven to reduce ovarian cancer mortality in the general population. This review is an update on the evidence behind ovarian cancer diagnostic strategies.

## 1. Introduction

Tubo-ovarian cancer (TOC) is the sixth most common cancer in females. It has an annual age-standardised incidence of 22.8 per 100,000 women, leading to a mortality rate of 12.2 per 100,000 women and accounting for 2% of total cancer deaths in the UK [1]. Its incidence is projected to increase by 5% over the next decade with an accompanying 15% decrease in mortality rate, reflecting population ageing and improved treatment strategies [2,3]. Screening trials have shown no impact on mortality and have documented harms: positive tests on asymptomatic women expose them to surgical risks for benign pelvic conditions, and early cancer cases may still be missed at screening. Consequently, to date, no test should be routinely used to screen women for ovarian cancer. There is a lack of pathognomonic symptoms of TOC at presentation, resulting in delays in diagnosis. Overall, 75% of patients are diagnosed at advanced stages of disease, which is associated with poorer prognosis. Serum biomarkers, risk models based on ultrasound and multimodal algorithms have improved diagnostic accuracy of adnexal masses and assessment of the risk of malignancy. This facilitates better patient counselling and appropriate referral to tertiary level oncology centres for further diagnostics, staging and possible cytoreductive surgery. The current review serves as an update on the current evidence on TOC diagnostic strategies.

## 2. Ovarian Cancer Diagnostic Pathways

TOC-related symptoms are non-specific and do not fit an easily recognisable pattern. This often leads to diagnostic delays which can be recognised at three main stages in the patients’ pathway: (1) the period from symptom onset to first clinical consultation (patient interval), (2) the period from primary review to specialist referral (primary care interval) and (3) the ‘secondary care interval’ from secondary care referral to diagnosis. Mendonca et al. showed that 36% of patients with TOC have more than three primary care consultations before being referred to a specialist, which compares poorly with other primary malignancies such as endometrial cancer [4].

**Figure 1:**
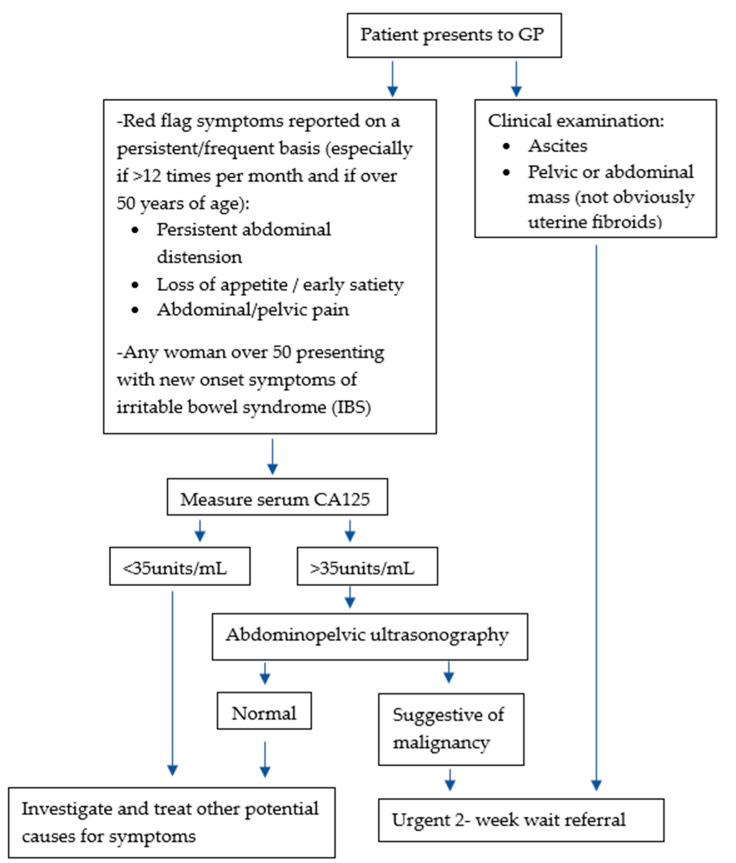
Ovarian cancer: recognition and initial management. Adapted from NICE Clinical guideline [5].

The National Institute for Health and Care Excellence (NICE) guidelines for referral of patients with suspected tubo-ovarian malignancy from primary care services divides diagnostic pathways based on the patient’s presenting symptoms [5] (Figure 1).

Patients presenting with signs or symptoms suggestive of ovarian cancer such as ascites or a pelvic mass are referred urgently to a specialist via the ‘two-week wait’ pathway. Patients with non-specific but concerning symptoms such as weight loss, abdominal pain or bloating undergo primary care testing, initially through CA125 serum testing. If CA125 is raised (≥35 units/mL), this is followed up with transvaginal pelvic ultrasonography. Two-week wait specialist referral is once again indicated if ultrasonography suggests a possible tubo-ovarian malignancy. Parallel testing strategies have also been proposed wherein patients undergo both serum CA125 testing and ultrasonography if symptomatic, as opposed to the interval sequential approach recommended by NICE. A third approach includes the use of multivariate algorithms such as age-based risk tools to guide referral.

The 2022 NHS England ‘Faster Diagnostic Standard’ has set a target for cancer to be refuted or confirmed within 28 days of GP referral [6]. This followed an initial 2015 recommendation by the Independent Cancer Taskforce report [7] which was reaffirmed in the 2019 NHS Long Term Plan [8]. The goal of this 2022 ‘Faster Diagnostic Standard’ is to diagnose cancer at an earlier stage when treatment may be more successful [6]. Through a reduction in secondary care interval delays, patients also experience less psychological distress. This represents a more patient-centred performance indicator over the ‘two-week wait’ to first appointment target and aims to reduce unwarranted variation across health service providers. Secondary care ‘one-stop clinics’ are a step in this direction.

## 3. Screening and Risk Reduction Interventions

Screening for TOC has been a subject of ongoing debate. Currently, there is no evidence to support TOC screening in either average or high-risk populations. In 2018, the United States Preventive Services Task Force (USPSTF) group reviewed evidence on risks and benefits for TOC screening in asymptomatic women and published a recommendation statement against screening for ovarian cancer in asymptomatic women who are not at high risk for the disease. In a meta-analysis, the group found that screening the general population conferred no mortality benefit and that there were moderate to substantial harms for patients having false-positive screening test results and subsequent surgery [9].

Among the largest conducted studies is the UK Collaborative Trial of Ovarian Cancer Screening (UKCTOCS) study, in which 202,638 healthy average-risk post-menopausal patients (50–74 years) were randomised to receive no screening, annual pelvic ultrasound, or multimodal screening using a combination of CA125 testing (interpreted using risk of ovarian cancer algorithm [ROCA]) and ultrasonography triggered by an increased ROCA score. Overall, the incidence of stage I or II disease was 39.2% higher in the multi-modal screening arm, whereas the incidence of advanced stage III/IV disease was 10.2% lower than that in the non-screening arm. After a median follow-up of 16.3 years, the authors showed this stage shift (downstage) did not translate into a significant survival benefit. This is the first trial to highlight the importance of specifying cancer mortality as the primary outcome in screening trials [10].

The US-based Prostate, Lung, Colorectal and Ovarian (PLCO) Cancer Screening Trial similarly included 78,216 healthy average-risk women between the ages of 55 and 74 years old who were randomised to have either annual CA125 testing for 6 years and transvaginal ultrasound examination for 4 years or a control arm without screening tests. After a median follow-up of 12.4 years, this study showed that using a baseline annual measurement of CA125 with a 35 U/mL cut-off in combination with pelvic ultrasound does not result in decreased ovarian cancer mortality [11].

The United Kingdom Familial Ovarian Cancer Screening Study (UK FOCSS) specifically looked at the role of multimodal screening (ROCA-based biomarker testing and transvaginal ultrasonography) in high-risk women with an estimated lifetime TOC risk of 10% or higher, secondary to familial cancer syndromes. Risk-reducing salpingo-oophorectomy (RRSO) was encouraged throughout the trial. After a median follow-up time of 4.8 years, the sensitivity for TOC detection within a year of screening was found to be 94.7% with a positive predictive value of 10.8% and a negative predictive value of 100%. There was lower incidence of Stage III-IV disease in patients within a year of screening (36.8%) when compared to patients diagnosed beyond a year from screening (94.4%). This study did not look at long-term mortality outcomes. The authors concluded that ROCA-based screening is an option for women with a familial cancer syndrome at increased TOC risk who defer or decline RRSO [12]. The ALDO project assessed the ‘real-world’ performance and cost-effectiveness of ROCA-based screening in women with pathogenic BRCA variants who defer or decline risk-reducing surgery. The authors reported that screening down-staged TOC at diagnosis led to a high complete cytoreduction rate (83%) and led to cost savings in the NHS setting (incremental cost-effectiveness ratio cost-saving of -GBP 102,496 per quality-adjusted life year) [13].

There is increasing evidence that the aetiopathogenesis of high grade serous ovarian cancer originates from the fallopian tube [14]. Around 7 in 10 precursor lesions (serous intra-epithelial carcinomas—STICs) are found in the tube and not the ovary, supporting this hypothesis of tubal origin of TOC. Therefore, researchers like Manchanda et al. have pioneered a two-step risk-reducing approach, where high-risk individuals (>4% risk for premenopausal women) are offered risk-reducing early salpingectomy (RRES) followed by delayed oophorectomy (DO) [15,16]. This two-step RRES-DO approach is currently practiced solely on the grounds of clinical trials in the UK, USA and Netherlands. The theoretical main advantage of the two-step approach is fewer menopause-related symptoms and hence higher acceptability and satisfaction, whilst maintaining generic quality of life; and indeed, the first data have confirmed this [17]. However, more robust data from ongoing trials are awaited, and this will delineate the exact oncological benefit as well as long term effect of RRES on menopause. On the other hand, there is wide use of opportunistic salpingectomies in average-risk individuals, as part of routine gynaecological surgery. Nevertheless, there is a paucity of evidence on the effect of opportunistic bilateral salpingectomy on menopause and on the long-term quality of life profile.

## 4. Pre-Test Risk Estimation Algorithms

Personalised, pre-test risk estimation algorithms play an important role in supporting decision making by women, as the perceived clinical risk level has been shown to increase the uptake of diagnostic tests and risk management strategies [18,19]. The majority of these tools are based on ‘red flag symptoms’, in combination with demographic or clinical variables to increase test sensitivity and specificity.

A widely used tool is the Goff symptom index. In their exploratory study, Goff et al. assessed 23 symptoms that are associated with TOC. Abdominopelvic pain, urinary urgency/frequency, bloating, and poor appetite/early satiety were found to be statistically correlated if they were present for less than a year and occurred for more than 12 days per month [20]. In their follow-up study, the Goff index had a 56.7% sensitivity for early-stage disease and 79.5% for advanced-stage disease. The specificity for women over 50 years was 90%, while for women under the age of 50, this was 86.7%. Investigators showed that depression and a negative affect were also higher in women who then went on to be diagnosed with cancer. These are thought to have an impact on how symptoms are reported as they were positively correlated with both the total number and frequency of reported symptoms.

Symptom indices have also been shown to correlate with poorer prognosis in postmenopausal women with ovarian cancer. Dilley et al. reported on 574 women who were diagnosed with invasive epithelial TOC in the ‘no screen’ UKCTOCS control arm comprising 101,299 women. At-risk symptomatic women identified using NICE guidelines or the modified Goff Index had significantly worse survival than those who were not, with each additional symptom at presentation decreasing overall survival [21].

Another risk prediction tool aimed for general practice is the ‘QCancer’ risk model [22]. The female version of the QCancer algorithm was developed on large primary care datasets and demonstrates good discrimination with a receiver operating characteristic (ROC) value of 0.84 for TOC. The model provides individualised risk values for different malignancies based on a combination of baseline risk factors (smoking, family history, body mass index, alcohol intake) as well as clinical symptoms. Using a 10% risk threshold to identify patients at higher risk for ovarian cancer, this tool has been shown to have a sensitivity of 64.1% and a specificity of 90.1%.

A disadvantage of symptom-based pre-test models is the significant inter-observer variability in the interpretation of patient-reported symptoms. This potentially leads to widely different cancer risk assessments and subsequent patient anxiety. Bankhead et al. highlighted the importance of careful history-taking for specific symptoms such as bloating, to include the timeline of symptom onset and whether this is fluctuant, persistent or progressive [23]. This may extend to other red-flag symptoms and how these are interpreted in the context of risk tool symptom checklists.

The increasing demand for genetic testing emphasises the growing need to improve the quality of individualised information disclosed with patients. Multifactorial risk prediction models can now provide personalised risk estimates for women with a positive family history of cancer or with confirmed pathogenic gene variants. One such risk algorithm is the CanRisk Tool, an online risk model that incorporates the latest version of the BOADICEA (Breast and Ovarian Analysis of Disease Incidence and Carrier Estimation Algorithm) [24]. This risk prediction tool incorporates a wide selection of variables including genetics, family history, polygenic risk scores as well as epidemiological risk factors.

There are challenges to the introduction of such personalised tools into consultations in the primary setting. There is research showing that a proportion of general practitioners are reluctant to reveal information about high individual cancer risks. Reports on the use of family history-based cancer risk assessment tools have also shown that if risk information is presented too explicitly, there is a potential to ‘lose control of the consultation’ [25].

## 5. Protein Diagnostics

One of the most widely used serum biomarkers for ovarian cancer is CA125, also known as Mucin-16(MUC16), which is a member of the mucin family glycoproteins [26]. CA125 is highly glycosylated and creates a hydrophilic, lubricating barrier on the female reproductive tract epithelia, respiratory tract as well as the ocular surface. It is elevated in approximately 80% of patients with epithelial non-mucinous ovarian carcinomas although it lacks sensitivity and specificity for early stages of the disease. CA125 is elevated in only half the cases of Stage I TOC and in 80–90% of advanced cases. Following TOC diagnosis, CA125 is also used to biochemically monitor response to treatment and to assess for possible disease recurrence [27].

Pregnancy, inflammatory processes and the presence of benign conditions such as leiomyomas, adenomyosis and endometriosis raise CA125 levels and reduce its positive predictive value for malignancy [28,29]. These conditions are more prevalent in premenopause, thus resulting in a higher sensitivity for CA125 use in postmenopausal women, in whom a level above 35 units/mL should raise suspicion and trigger further investigations [26]. CA125 levels may also be influenced by a raised body mass index and ethnicity [30].

The combination of a more extended serum biomarker profile, including the addition of carbohydrate antigen CA 19-9 and carcinoembryonic antigen (CEA) to CA125, is useful for differentiating between gastrointestinal tract or hepatobiliary primaries and tubo-ovarian malignancy [31,32]. CEA is a protein normally found in embryonic and foetal tissue. Serum levels disappear almost completely after birth, but small amounts may persist in the colon. Raised levels (above 3.8 mcg/L) may be associated with adenocarcinoma of the colon.

A CA125 to CEA ratio greater than 25 is generally consistent with primary tubo-ovarian origin of a tumour. Both CA19-9 and CEA may, however, be raised with primary mucinous carcinoma of the ovary, which is a rare entity, representing only 3–4% of ovarian cancer diagnoses [33]. Raised CA19-9 and CEA levels and the diagnosis of intestinal-type mucinous ovarian pathologies therefore often prompt endoscopic assessment of the gastrointestinal tract to rule out a gastrointestinal primary with secondary Krukenberg deposits on the ovaries.

Multiple studies have attempted to combine other tumour markers such as Human epididymis protein 4 (HE4) with CA125 to reduce false-positive diagnostic tests in the presence of benign and inflammatory lesions in younger women [34,35,36]. HE4 is a glycoprotein produced in normal glandular epithelium of the reproductive tract, respiratory tract epithelium and renal tubules. Like CA125, serum HE4 levels are a useful preoperative test for predicting the nature of pelvic masses. Unlike CA125 however, HE4 is not increased in benign gynaecologic disease [37]; however, its concentration may be influenced by kidney function, smoking and age [38,39].

**Figure 2:**
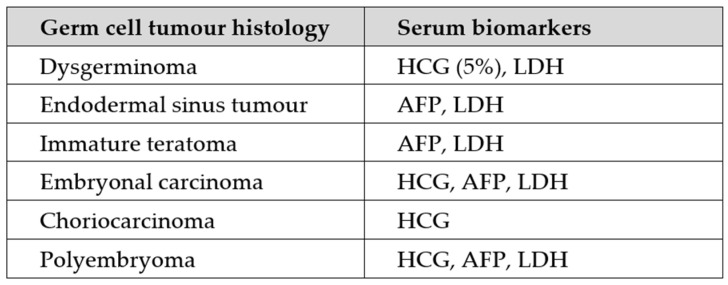
Serum biomarker profile associated with specific germ cell tumour histological subtypes adapted from Benoit et al. [41].

Diagnostics in younger patients with complex adnexal lesions vary in that these are at higher risk of germ cell tumours. Ovarian germ cell tumours arise from primordial germ cells of the ovary and often produce serum proteins which are used as a highly sensitive marker for the presence of specific histologic components (Figure 2). The Joint RCOG/BSGE guideline for the management of suspected ovarian masses in premenopausal women suggest that alpha-fetoprotein (α-FP), lactate dehydrogenase (LDH) and beta human chorionic gonadotropin (β-hCG) should be measured in all women under the age of 40 with a complex ovarian mass due to the higher prevalence of germ cell tumours in younger women [40].

Another non-epithelial group of ovarian tumours that are more prevalent in younger patients are sex cord–stromal tumours (SCST). These may produce sex hormones such as androgens and oestrogen that may cause secondary symptoms such as androgenising effects and menstrual cycle irregularities. Suppression of the normal follicle-stimulating hormone response to gonadotrophic-releasing hormone pulses via tumour-derived inhibin B may also lead to secondary amenorrhea [42,43]. In the female menstrual cycle, the inhibin B glycoprotein prevents further recruitment and growth of follicles close to ovulation. The production of oestrogen by granulosa cell tumours may also cause proliferative and hyperplastic changes in the endometrium potentially leading to type 1 endometrial carcinoma. The ESGO-SIOPe guidelines for the management of non-epithelial ovarian cancers in adolescents and young adults therefore recommend the measurement of serum AFP, β-hCG, CA125, Inhibin B, antimullerian hormone (AMH), and LDH in young patients with suspected sex cord–stromal tumours [44]. A hormone profile including oestrogen, dehydroepiandrosterone, testosterone, luteinizing hormone and follicle stimulating hormone is also advised.

## 6. Biomarker-Based Algorithms

Serum biomarkers have been incorporated into multivariate panels and algorithms in order to improve their sensitivity and predictive value. Strategies using combined biomarker-based tools have been shown to be more effective than single biomarker measurements at fixed thresholds. The majority of these assays are licensed to assess the risk of TOC; however, they are not intended to be used as a screening test.

### 6.1. Risk of Malignancy Index (RMI)

Perhaps the most widely used algorithm in the preoperative work up of suspected ovarian malignancy is the risk of malignancy index (RMI), which was proposed by Jacobs et al. in 1990 [45]. RMI employs a simple formula (RMI = M × U × CA125), taking into account menopausal status (M), findings on transvaginal ultrasound imaging (U) and serum CA125 level. The NICE guideline on the recognition and initial management of ovarian cancer recommends that people with an RMI-1 score of 250 or more should be referred to a specialist MDT [5]. The Royal College of Obstetricians and Gynaecologists recommends an RMI-1 threshold of 200 to predict the likelihood of TOC (Sensitivity 78%, specificity 87%) [46]. However, it recognises that some centres utilise an equally acceptable threshold of 250 with a lower sensitivity (70%) but higher specificity (90%). Patients with an RMI score of more than 200 have a 42-fold increased risk of TOC over the background population [45].

Since its initial development, four variations of the RMI algorithm have been proposed which alter the scoring system with regard to menopausal status and ultrasonographic findings (RMI-2 and RMI-3), or incorporate supplementary variables such as lesion size (RMI-4) or Doppler assessment of adnexal mass vascularity (RMI-5). While these versions were developed to improve sensitivity and specificity, comparative analyses have shown no significant practice-changing difference in the performance of these algorithms [47]. The ease of use of RMI-1 and its relatively low cost have facilitated clinical implementation in both the community and specialised centres.

### 6.2. Risk of Malignancy Algorithm (ROMA^®^)

Another biomarker assay for preoperative adnexal mass assessment is the Ovarian Risk of Malignancy Algorithm (ROMA^®^). This predictive index combines the serum measurements of HE4 and CA125 with menopausal status to stratify patients into low- or high-risk categories. It does not include ultrasound findings in the risk calculation. ROMA^®^ has been shown to have high sensitivity in distinguishing ovarian cancer in women with abnormal ultrasound findings. It has a sensitivity of 93.8% and a moderate specificity of 74.9% [48]. The additional specificity conferred by the inclusion of HE4 improves its ability to identify premenopausal malignancies [49]. This is because HE4 has a higher specificity than CA125 in distinguishing between malignant and benign gynaecologic diseases [50].

### 6.3. Copenhagen Index

The Copenhagen index also provides risk stratification based on serum CA125 and HE4 levels, in this case combined with patient age. Carreras Dieguez et al. reported that in postmenopausal women, and women with Stage I lesions, the Copenhagen index and ROMA^®^ outperformed HE4 and CA125 as a single test [51]. In 2023, Huiling Liu performed a meta-analysis to show that this index has a sensitivity of 82% and a specificity of 88% [52]. The advantage conferred by this algorithm is that it is independent of transvaginal imaging findings, and could thus be employed in places with limited access to ultrasonography.

### 6.4. OVA1^®^ and OVERA^®^

Another multivariate assay is OVA1^®^. This algorithm incorporates ultrasonographic findings, menopausal status and five serum biomarkers (CA125, apolipoprotein A1, transferrin, transthyretin and beta-2 microglobulin). It stratifies patients using a risk score with a range of 0–10 [53]. Following its inception, the OVA1^®^ test led to the development of a second-generation model called OVERA^®^ [54]. This incorporates three of the original OVA1^®^ serum markers (CA125, apolipoprotein A1, Transferrin) and uses HE4 and follicle-stimulating hormone instead of Transthyretin and B2-microglobulin. OVERA^®^ maintains the high diagnostic sensitivity of OVA1 (94%, 92%, respectively) while improving on specificity (65%, 42%, respectively).

### 6.5. Risk of Ovarian Cancer Algorithm (ROCA)

In practice, clinicians have also traditionally given a lot of weight to the trend in serum biomarker levels. This is especially so when initial diagnostics reveal inconclusive results and/or borderline serum biomarker levels. Olleg Blyuss et al. analysed two serial algorithms (method of mean trends and parametric empirical Bayes) to show the superiority of longitudinal algorithms compared to single-threshold rules [55]. The results emphasised the importance of incorporating serial biomarker level changes in early TOC detection strategies. A rapid rise in serum marker levels is given more significance as this may represent an ongoing malignant process with tumour growth. This was incorporated into the ROCA (Risk of Ovarian Cancer Algorithm) test, which was employed in the UKCTOCS trial.

## 7. Liquid Biopsies

An emerging diagnostic technique involves liquid biopsies that measure circulating tumour DNA (ctDNA), cell-free RNA (cfRNA), exosomes, tumour-educated platelets and circulating tumour cells in the blood. In a drive towards earlier diagnosis, studies have highlighted the diagnostic potential of ctDNA methylation status. Promoter methylation leads to epigenetic inactivation of tumour suppressor genes that plays an important role in carcinogenesis. In a cohort of 43 controls with benign ovarian tumours and 71 patients with epithelial tubo-ovarian carcinoma, Wang et al. showed that specific cell-free DNA (cfDNA) methylation was able to distinguish patients with early-stage TOC from healthy subjects [56]. Hypermethylation of the slit homologue 2 tumour suppressor gene has also been shown to represent an early event in the pathogenesis of TOC [57].

There is limited in vivo evidence for liquid biopsy use in clinical settings especially in the pre-symptomatic stage of TOC. Widschwendter et al. undertook a study using methylation analysis of the epigenetic markers C2CD4D, COL23A1, and WNT6 in the early screening setting and reported that they were able to detect TOC up to 2 years before clinical diagnosis with a sensitivity of 23% and specificity of 97% [58]. Li Boxuan et al. reported on a meta-analysis which showed that cfDNA appears to be similar to HE4 and slightly superior to CA125 in its diagnostic ability to distinguish between TOC patients and controls [59]. The authors emphasised that these results should be interpreted with caution and that further longitudinal studies need to validate the diagnostic potential of cfDNA before use outside of a research context.

There are several challenges that need to be addressed before routine clinical use of liquid biopsies, including test costs as well as limited sensitivity for early-stage ovarian cancer. Due to the low proportion of ctDNA in cell-free DNA, current isolation strategies need to be improved to enhance the extraction yield and enable ctDNA detection at low allele frequencies, thereby improving test sensitivity.

## 8. Imaging Diagnostics

Transvaginal pelvic ultrasonography is the most valuable initial tool and is the first line imaging modality for adnexal mass assessment. Additional transabdominal views are essential for large masses that extend out of the pelvis. The diagnostic accuracy of ultrasonography in distinguishing between benign and malignant adnexal masses correlates heavily with operator experience and expertise [60].

Assessment by expert operators has excellent performance when differentiating between benign and malignant ovarian tumours. This is based on subjective evaluation of the grey-scale ultrasound image with or without vascular flow assessment using colour Doppler examination [61]. The International Ovarian Tumour Analysis (IOTA) group defined terminology that standardises the description and documentation of sonographic features for ovarian masses [62]. The sensitivity of pattern recognition ranges from 88% to 98%, while specificity ranges from 89% to 96% [63,64]. However, not all centres have access to expert sonographers with enough case volume to maintain this standard. For this reason, multiple ultrasound-based diagnostic models have been developed to assist clinicians in risk of malignancy assessment by ultrasonography.

### 8.1. The IOTA Algorithms

Algorithms developed by the IOTA group comprise methods that are computer-independent such as the IOTA simple rules and the IOTA benign descriptors, as well as methods that require computer-based mathematical modelling such as the logistic regression models L1 and L2 and the ADNEX (Assessment of Different NEoplasias in the adneXa) model. All IOTA methods have been prospectively validated on large cohorts and shown have excellent discriminative ability, outperforming RMI and ROMA [65,66].

**Figure 3:**
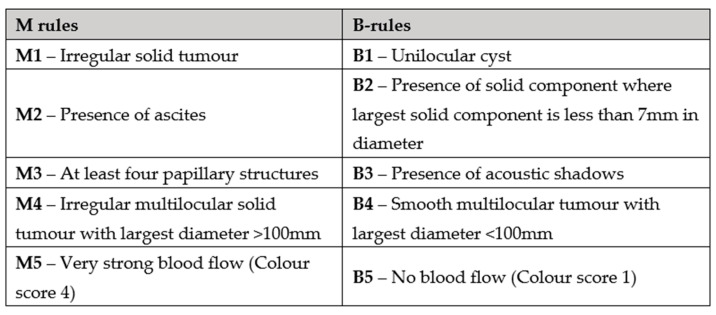
IOTA simple rules comprising B (benign) and M (malignant) features [67].

The IOTA simple rules were developed in 2008 and consist of a set of five findings predicting a benign tumour (B-features) and five findings predicting a malignant tumour (M-features) [67] (Figure 3).

If one or more M features are identified in the absence of a B feature, the mass is categorised as being malignant (Rule 1). If one or more B features are identified in the absence of an M feature, the mass is then classified as being likely benign (Rule 2). If both or neither of the rules are present, the evaluation is considered inconclusive or indeterminate (Rule 3). Nunes et al. showed that the IOTA simple rules were able to classify 76–89% of adnexal masses as benign or malignant with a sensitivity of 93% and a specificity of 90% [68]. In a cross-sectional cohort study involving 5020 patients, Timmerman et al. confirmed the diagnostic performance of the simple rules across 22 centres including oncology centres, referral centres for ultrasonography, and general hospitals [69]. In patients deemed to have a higher risk (>30%) of malignancy, the sensitivity of these rules was 89.0% with a specificity of 84.7%. The method performed well at all levels of care provision, confirming its relative ease of use and implementation.

**Figure 4:**
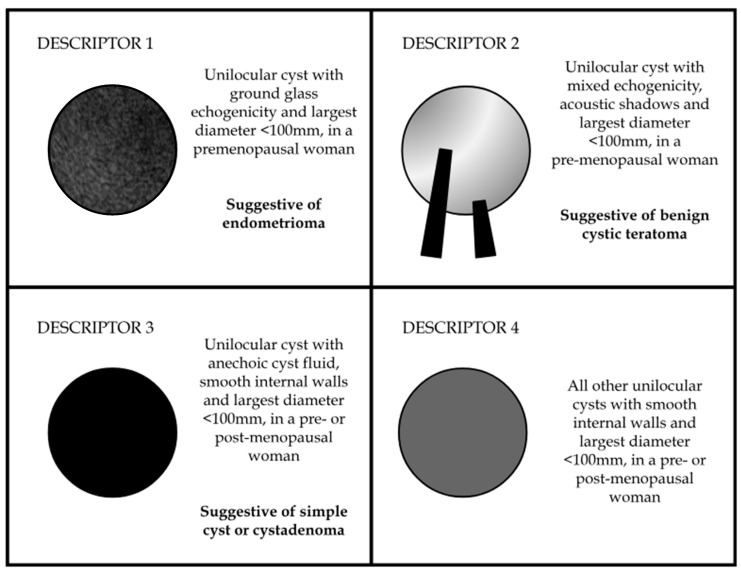
Benign simple descriptors for the identification of benign ovarian cystic lesions.

The IOTA modified ‘easy descriptors’ consist of four criteria based on clinical and ultrasound information that provide a rapid diagnosis of a benign adnexal mass. These descriptors reflect the fact that pattern recognition is a key part of ultrasonography and were adapted from six ‘instant’ descriptors first described in 2012 [70]. The four modified benign simple descriptors (Figure 4) include (1) a unilocular tumour with ground glass echogenicity and largest diameter < 100 mm in premenopausal women suggestive of an endometrioma; (2) unilocular cyst with mixed echogenicity, acoustic shadows and largest diameter < 100 mm in a premenopausal woman—suggestive of benign cystic teratoma; (3) unilocular anechoic cyst, with smooth internal walls and largest diameter < 100 mm in a pre- or post-menopausal woman—suggestive of simple cyst or cystadenoma; and (4) remaining unilocular cysts with smooth internal walls and largest diameter < 100 mm in a pre- or post-menopausal woman [71]. Around 43% of all adnexal lesions could be ‘instantly’ diagnosed with the simple descriptors. If a modified benign simple descriptor can be applied to an adnexal mass, the tumour is almost certainly benign—the risk of malignancy is low at <1% [72]. Similarly, two ‘easy’ malignant descriptors were described: (1) the presence of a tumour with ascites and at least moderate Doppler vascular flow in the post menopause, and (2) the presence of an adnexal lesion in females above the age of 50 with a CA125 level above 100 units/mL.

Logistic regression (LR) models, LR1 and LR2, were then developed as a further diagnostic tool. The original LR1 model has 12 variables comprising a combination of demographic variables (such as age), clinical factors (such as family history and hormone therapy) as well as the adnexal ultrasound features. A simpler model (LR2) uses only six variables from the LR1 model [73]. Using a risk cut-off of 10%, the LR2 model has been shown to have a sensitivity of 92% and specificity of 83% in differentiating benign from malignant adnexal masses, outperforming the RMI model [65].

The ADNEX model was developed in 2014 [74]. The output from this validated model is not limited to differentiation between benign and malignant lesions. It can preoperatively quote the percentage risk of benign, borderline, early (Stage I) or advanced (Stage II-IV) invasive disease, as well as secondary metastatic ovarian deposits. It is a computer-based model that comprises three clinical and six ultrasound predictors: age, serum CA125 level, centre type, maximum lesion diameter, proportion of solid tissue, number of cyst locules (more than 10), number of papillary projections, acoustic shadows, and ascites. Although CA125 is not a mandatory variable to the model, it improves test performance. Several external validation studies have shown excellent performance with an area under the curve (AUC) ranging between 0.91 and 0.97 for basic discrimination of benign and malignant tumours [75,76]. The 2021 ESGO/ISUOG/IOTA/ESGE consensus statement on pre-operative diagnosis of ovarian tumours states that if an adnexal mass has a more than 10% risk of malignancy, a referral to a specialised gynaecological oncology centre should be made. Surgical management in a general gynaecological unit may be advised for lesions with low risk (1–10%) of malignancy, while conservative management is safe for lesions that are most certainly benign (<1% risk) [77].

Multi-step strategies have been described to facilitate the integration of these models into clinical practice. A three-step strategy was proposed in 2012 using the benign and malignant simple descriptors as a first step, followed by the IOTA simple rules as a second step and by subjective expert assessment as a final step should interpretation still remain inconclusive [70]. This approach has excellent discriminative ability for malignant lesions; however, it relies on the presence of an expert sonographer, which is not always the case. A two-step strategy was therefore developed in 2022 [71]. This strategy recommends the application of benign simple descriptors as a primary step. If a benign simple descriptor can be applied, the mass is almost certainly benign (<1% risk of malignancy). If this assessment is inconclusive, the second step would then involve using the ADNEX model. The two-step strategy has an AUC of 0.93 for discriminating between a benign and malignant tumour. Using the ADNEX model on all masses has no advantage over using the two-step strategy.

Additionally, ultrasound has been shown to have good diagnostic performance in the assessment of ovarian cancer spread in the abdomen [78]. The ISAAC study showed that in experienced hands, ultrasound performs better than standard computed tomography (CT) and can be an alternative technique to magnetic resonance imaging with diffusion-weighted imaging (DWI MRI) in the evaluation of peritoneal carcinomatosis in advanced ovarian cancer. It had a higher sensitivity for the detection of pelvic peritoneal involvement, deep rectosigmoid wall infiltration, parenchymal liver disease, as well as lesser and greater omental deposits and anterior abdominal wall lesions. All three modalities performed similarly in retroperitoneal lymph node staging [79].

### 8.2. Magnetic Resonance Imaging (MRI)

**Figure 5:**
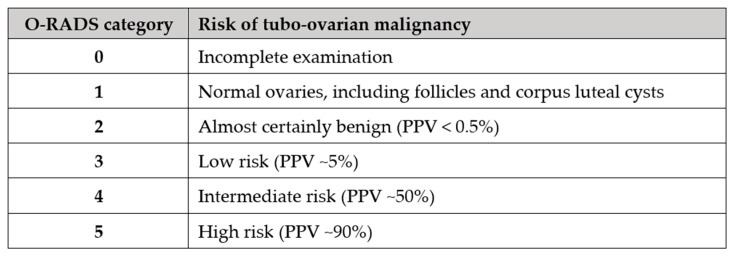
The O-RADS MRI categories and their respective interpretation.

Magnetic resonance imaging (MRI) including functional sequences, dynamic contrast-enhanced and diffusion-weighted imaging may be used as a second-line tool after ultrasonography to further differentiate between benign, malignant, and borderline masses [80]. The administration of gadolinium contrast is essential because it may reveal solid components not appreciated on the pre-contrast T1- and T2-weighted images. The Ovarian-Adnexal Reporting and Data System MRI risk score (O-RADS) is a stratification system that allows radiologists to assign the probability of malignancy to an adnexal mass based on the composition of the lesion, the signal intensity characteristics, and the enhancement pattern of any solid tissue. There are six risk score categories in the O-RADS MRI risk stratification system (Figure 5).

Studies evaluating the performance of IOTA models (LR2 and ADNEX) as compared to the subjective interpretation of MRI findings by experienced radiologists have favoured the ultrasonography-based models for greater specificity and better accuracy at diagnosing borderline ovarian tumours [81,82].

Diffusion-weighted magnetic resonance imaging (DWI) is a technique which enables refined tumour characterisation by depicting the restricted movement of water molecules within hypercellular malignant tumours. It not only improves primary tumour characterisation, but yields better sensitivity than CT and positron emission tomography (PET-CT) for peritoneal staging and improves the detection of peritoneal nodules and lymph node metastases [83]. DWI MRI has been shown to be significantly more accurate than CT in detecting disease burden in critical potentially unresectable areas, such as mesenteric carcinomatosis and large bowel carcinomatosis, allowing for prediction of residual disease after cytoreductive surgery [84]. However, this technique has some limitations such as longer acquisition times and motion sensitivity.

### 8.3. Computed Tomography (CT)

Computed tomography’s main role in ovarian cancer diagnostics is in the staging and evaluation of disease distribution in cases with high suspicion of TOC. It also plays a role in detecting primary tumours of non-ovarian origin that may present with similar signs and symptoms due to metastatic ovarian deposits. CT protocols with standardised carcinomatosis index forms are used to assess the extent of tumour dissemination during radiological staging and surgical planning [85,86]. When neoadjuvant chemotherapy is offered, an imaging-guided biopsy, planned on the staging CT, confirms the diagnosis and provides the histological sub-type. As described by Sahdev et al., a systematic CT reporting approach is essential to identify and communicate important sites of disease which may alter or preclude surgery at diagnosis or following neoadjuvant chemotherapy [87]. Quantifying the extent of bowel serosal and mesenteric deposits is technically difficult to achieve and communicate. One of the limitations of CT is its inability to demonstrate small volume (<5 mm) deposits on the bowel serosa, mesentery and peritoneum. Evaluation may sometimes necessitate laparoscopic assessment to further assess disease distribution and resectability. Despite these limitations, with good imaging acquisition and interpretation, CT remains excellent with reported accuracies of 70–90% for detection of disease at all stages [88].

### 8.4. Fluorodeoxyglucose Positron Emission Tomography (FDG-PET)

FDG-PET CT has a high positive predictive value for diagnosing primary as well as recurrent ovarian cancer. It can therefore be useful in differentiating malignant from borderline or benign ovarian tumours; however, its performance can be impacted negatively by reduced fluorodeoxyglucose uptake in clear cell and mucinous invasive subtypes [89]. PET-CT cannot differentiate reliably between borderline and benign tumours and therefore plays no role in the context of considering fertility-sparing interventions. This imaging modality is often used for problem-solving, particularly to characterise unclear lesions in key potentially unresectable areas that would significantly alter clinical management (such as thoracic lesions) [90,91]. Another limit of FDG-PET CT is the false-negatives rate observed with small metastatic lesions (less than 10 mm and particularly less than 7 mm) [92]. Areas or processes with high metabolic activity may also lead to false positive findings. These include infection, inflammation as well as physiologic muscle and renal activity [93].

## 9. Computer-Aided Diagnostics and Machine Learning

Computer-aided diagnostics and artificial intelligence are playing an ever-increasing role in clinical research and management. Artificial intelligence has been shown to support and enhance diagnostics, refine decision making as well as support individualised treatment strategies for gynaecological malignancies. Within the field of gynaecologic oncology, however, it is a relatively new concept compared to other medical specialties such as pathology, dermatology and neurology, where it is being adopted into clinical practice [94,95,96]. Definitive diagnosis of ovarian tumours requires either surgical resection or invasive image-guided biopsies and histological evaluation. Surgical decision making is at times challenging especially with atypical or inconclusive pre-operative findings and in cases where fertility-preservation is desired. Artificial intelligence has the potential to combine and interpret granular objective variables to provide clinicians with a numerical value relating to the risk of malignancy. This could in turn prevent unnecessary surgeries or treatment radicality, and support case prioritisation.

Tools for automated imaging analysis are being developed to reduce the significant interobserver variability that is present in the assessment of malignancy risk and disease resectability [97]. In the era of big data science and machine learning, a new field in medical imaging, radiomics, has emerged. Radiomics is a quantitative approach to imaging, which aims to improve image interpretation through mathematical computations. The process encompasses (a) image acquisition and segmentation, (b) feature extraction, (c) feature selection and (d) model construction [98]. Textural scan information can be inferred through signal intensity quantification and pixel interrelationships, and subsequent application of artificial intelligence methods.

Song et al. evaluated predictive radiomics models on 104 ovarian lesions using data extracted from magnetic resonance imaging. They analysed the predictive capability for three tasks: distinguishing benign from borderline lesions (task A), from malignant lesions (task B), and distinguishing between borderline and malignant masses (task C) [99]. The radiomics model showed a good diagnostic capability with an area under the curve (AUC) of 0.899, 0.865, and 0.893, respectively. Similarly, Zhang et al. evaluated the diagnostic performance of a radiomics model based on MR images using features from multiple sequences. The diagnostic performance of MR radiomics was superior to that of radiologists at discriminating benign ovarian tumours from malignancies (90.6% and 83.5%, respectively). The most common pitfall among traditional sequence interpretation of MR among radiologists was the classification of borderline ovarian tumours into the benign group. Diagnostic performance of radiologists was in fact non-inferior to the radiomics model when borderline tumours were excluded from the analysis [100].

The role for artificial intelligence is not limited to cross sectional imaging. Aramendía-Vidaurreta et al. applied a novel neural network approach for the automatic discrimination of adnexal masses based on ultrasonographic images and patients’ age. This technique analysed seven different ultrasonographic variables from adnexal masses in 145 patients and resulted in a sensitivity of 98.50% and a specificity of 98.90% [101]. Similar techniques have also been applied to serum biomarkers. Kawakami et al. used seven machine learning classifiers on 32 commonly available pre-operative parameters as well as age for 334 patients with epithelial ovarian cancer and 101 patients with benign ovarian tumours. Machine learning correctly discriminated between benign and malignant lesions in 92.4% of cases, and could also correctly predict high grade serous and mucinous histological subtypes [102]. Gu et al. also interestingly used a support vector machine-based machine learning algorithm to show that there are subtle postprandial serum CA125 level changes that are more pronounced with malignant ovarian pathologies. Using 1 h post prandial CA125 profiles, the algorithm was shown to have 91.7% sensitivity and 99.2% specificity [103].

## 10. Conclusions

The focus of ovarian cancer diagnostic strategies is shifting towards earlier diagnosis of the disease. Early recognition of red flag symptoms and coordinated patient flow along the diagnostic and treatment pathway from the community to specialised cancer centres, is essential to improve patient outcomes. Thorough history taking and clinical examination remain indispensable for the assessment of suspicious symptoms. Pre-test risk estimation algorithms based on cancer-related risk variables and symptom assessment are a valuable individualised supplement for these first consultations.

CA125 serum testing remains the most widely adopted first-line investigation for patients with suspected ovarian cancer. Longitudinal biomarker trend assessment, multivariate panels and biomarker-based algorithms have consistently been shown to have better sensitivity and specificity than CA125 alone in TOC detection. Ultrasound remains the first-line imaging modality in ovarian cancer diagnostics, and its use is becoming less reliant on the availability of expert high-volume practitioners following the introduction of the two-step strategy by the IOTA group.

In the future, novel diagnostic approaches such as circulating tumour DNA and cell-free RNA assessment as well as methylation analysis may provide an opportunity for TOC screening and for its detection at an earlier stage. Artificial intelligence and machine learning hold promise for improving objective radiological risk stratification of adnexal masses. The application of these novel techniques into clinical practice is likely to face several challenges including financial costs, training requirements and impact on laboratory workloads.

## Data Availability

Data sharing not applicable.
